# CD147/Basigin: From Integrative Molecular Hub to Translational Therapeutic Target

**DOI:** 10.1002/advs.202518884

**Published:** 2025-12-27

**Authors:** Xiang‐Min Yang, Huijie Bian, Zhi‐Nan Chen

**Affiliations:** ^1^ Department of Cell Biology National Translational Science Center for Molecular Medicine The Fourth Military Medical University Xi'an, Shaanxi 710032 China; ^2^ State Key Laboratory of New Targets Discovery and Drug Development for Major Diseases Xi'an, Shaanxi 710032 China

**Keywords:** Autoimmune diseases, CD147, Infectious diseases, Matrix metalloproteinase (MMP), Tumor microenvironment

## Abstract

CD147 (Basigin/EMMPRIN), a multifunctional member of the immunoglobulin superfamily (IgSF), is a critical regulator of tumor progression, immune modulation, and metabolic adaptation. Under physiological conditions, it acts as a dynamic scaffold, interacting with monocarboxylate transporters (MCTs), integrins, and cyclophilin A (CyPA) to orchestrate spermatogenesis, embryo implantation, and neural network function. Pathological overexpression of CD147 induces the secretion of matrix metalloproteinases (MMPs), epithelial‐mesenchymal transition (EMT), metabolic reprogramming, and immune evasion, functioning as an independent prognostic biomarker in multiple malignancies. Beyond oncology, CD147 is exploited as an entry receptor for pathogens, including SARS‑CoV‑2, HIV‑1, *Plasmodium falciparum*, and contributes mechanistically to cardiovascular, autoimmune, and neurodegenerative diseases. Notably, CD147 acts as a fundamental “Energy‐Structure Coupler,” coordinating metabolic flux (via MCTs) with morphogenetic plasticity (via integrins/MMPs) to maintain cellular homeostasis. This review summarizes current insights into CD147's molecular structure, isoforms, post‐translational modifications, and signaling pathways, highlighting its pivotal roles across cancer, infection, autoimmunity, and cardiovascular disease. Finally, we discuss challenges such as the “specificity paradox” and propose emerging strategies to exploit CD147 as a precision biomarker and therapeutic target across diverse diseases.

## Graphical abstract

This review conceptualizes CD147 as a fundamental “Energy‐Structure Coupler,” physically bridging metabolic flux (via MCTs) with morphogenetic plasticity (via integrins/MMPs) to drive cancer, infection, and autoimmunity. Addressing the “specificity paradox” that limits current translation, the authors chart a strategic roadmap—spanning logic‐gated therapies to precision biomarkers—transforming this multifunctional hub into a viable target for next‐generation medicine.

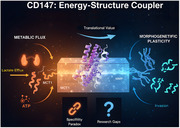

## 1 Introduction

Since its initial description by Biswas et al. in 1982 as the tumor cell–derived collagenase stimulatory factor (TCSF) [1, 2], CD147 (Basigin/EMMPRIN) has evolved into a multifunctional transmembrane glycoprotein that governs matrix remodeling [3, 4], microenvironmental communication [5, 6] and, more recently, metabolic [7] and immune regulation [8, 9]. As a structurally dynamic and functionally versatile member of the immunoglobulin superfamily (IgSF) [10, 11], CD147 serves as a molecular hub orchestrating physiological homeostasis [12] and a wide range of pathological processes, including tumor progression [13], pathogen invasion [14], immune modulation [15], and metabolic dysregulation [8, 16]. Structurally, CD147 comprises two extracellular immunoglobulin‐like domains containing glycosylation sites (Asn44, Asn152 [17], Asn186) that regulate conformational stability and ligand affinity [18]. Its transmembrane region contains a leucine zipper motif that enables homo‐dimerization [19, 20], while the intracellular domain activates critical signaling pathways, including PI3K/Akt and MAPK [21, 22]. The functional diversity of CD147 is further amplified by tissue‐specific splice variants (Basigin‐1 to ‐4) [23] and post‐translational modifications [24], such as glycosylation [18] and methylation [25, 26, 27], thereby enhancing its adaptability to diverse biological environments [28, 29, 30].

Over the past four decades, the conceptual landscape of CD147 has shifted profoundly—from a singular matrix regulator [31, 32, 33] to a pivotal “Energy‐Structure Coupler” that bridges metabolic flux, immune response, and tissue remodeling [34, 35, 36, 37]. However, despite its established prominence in oncology and infectious diseases, critical knowledge gaps remain [38]. These include the dynamic switching between its metabolic versus proteolytic functions [39, 40], limited clinical translation, concerns over off‑target effects [28, 41], and insufficient investigation in neurological disorders [28, 29, 30]. Additional underexplored areas involve its subcellular localization (e.g., mitochondria, extracellular vesicles [42, 43]), and the influence of genetic polymorphisms (e.g., rs8259 SNP) on drug response [44, 45].

This review systematically evaluates four decades of CD147 research, organized into three thematic sections: (1) Mechanistic insights: detailing structural variants, post‐translational regulation, and intracellular signaling (including CD147‐ICD–mediated nuclear transcription [46]); (2) Disease integration: connecting roles in cancer, infection, cardiovascular disease [47], and autoimmunity through the perspectives of “Energy‐Structure Coupling” [13, 48]; and (3) Translational roadmap: assessing current therapeutic strategies (mAbs, CAR‐T) and outlining future directions involving spatial omics [49, 50] and logic‐gated precision targeting [51, 52]. By presenting a multidimensional functional landscape, this review aims to bridge foundational biology with clinical innovation, advancing CD147 from a molecular concept to a cross‐disease therapeutic paradigm.

## 2 Discovery History and Milestones of CD147 Research

**FIGURE 1:**
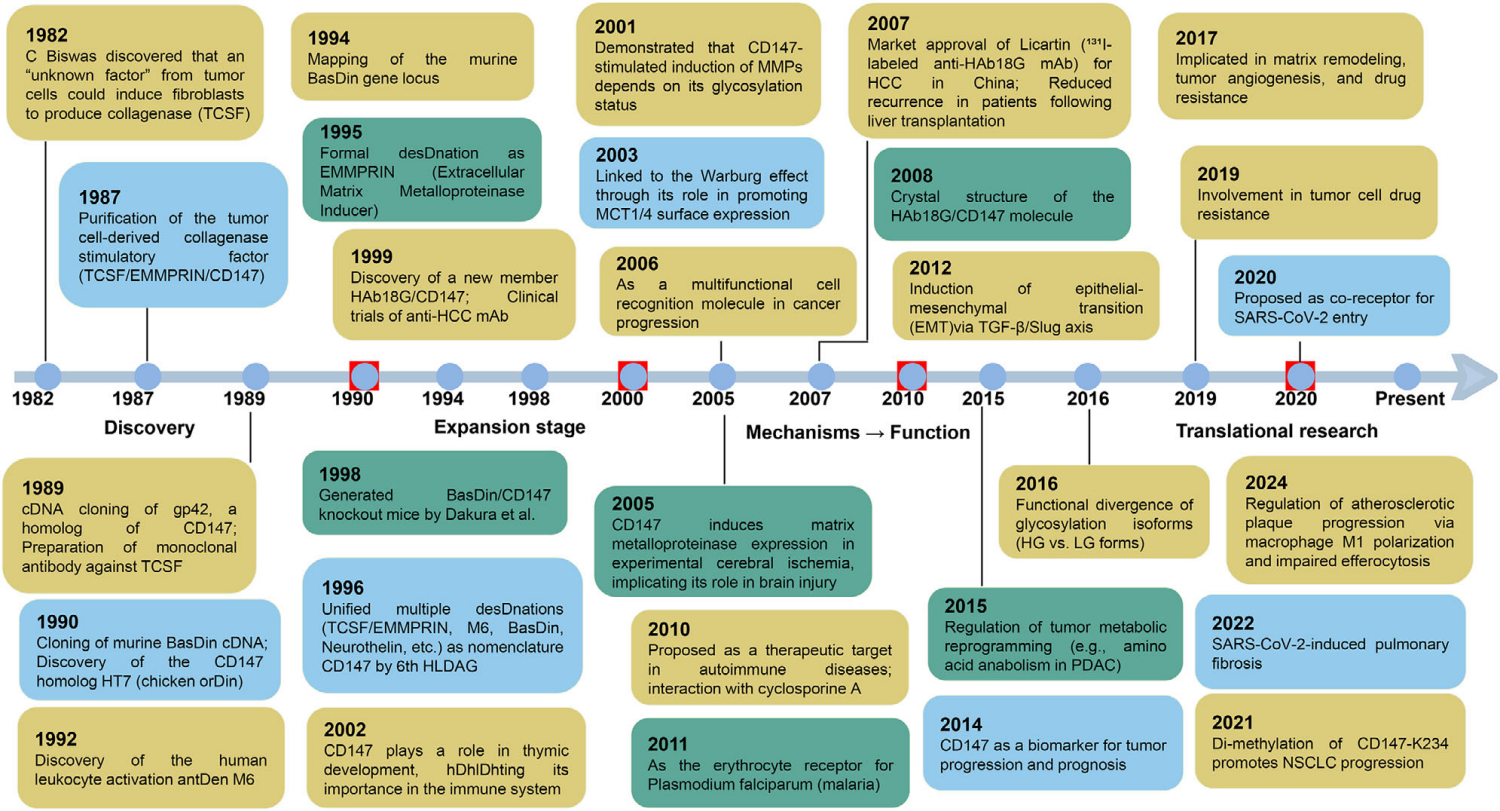
Chronological map of CD147 research: from discovery to translational applications. This timeline traces the four‐decade research of CD147 (Basigin/EMMPRIN) from 1982 to present, spanning its trajectory through discovery, mechanistic elucidation, and translational development. Key phases include the initial identification as a tumor‑derived collagenase stimulatory factor (TCSF), the elucidation of its structural biology, and its recognition as a central hub in cancer, metabolic regulation, and infectious diseases (e.g., malaria and COVID‑19). Pivotal events include the market approval of Licartin in 2003, structural elucidation in 2008, and identification as a proposed SARS‑CoV‑2 co‑receptor in 2020, underscoring its evolving significance in biomedical science.

CD147, known by aliases such as tumor cell‐derived collagenase stimulatory factor (TCSF), extracellular matrix metalloproteinase inducer (EMMPRIN) [6, 53], leukocyte activation antigen M6 [54], Basigin/gp42 (BSG) [55, 56], HAb18G/CD147 [57], Neurothelin [58], OX‐47/CE‐9 (rat) [54], HT7/5A11 (chicken) [58], Ok blood group [59] and others, reflects its pleiotropic roles across species and tissues. First identified in 1982 as TCSF by Biswas [1], it was renamed EMMPRIN in 1987 [31, 60]. Crucially, the HAb18G/CD147 variant was identified by screening a hepatocellular carcinoma cDNA library with the monoclonal antibody HAb18 [57, 61]; sequence analysis confirmed it shares an identical open reading frame with CD147, establishing its identity as a unified molecular entity within the superfamily [62]. Subsequently, the designation “CD147” was formalized at the Sixth Human Leukocyte Differentiation Antigen (HLDA) Workshop to unify nomenclature, while the Human Gene Nomenclature Organization (HUGO) [63] standardized the gene name as *Basigin* [63], ensuring consistency in biomedical research (Figure 1). As a ubiquitously expressed member of the immunoglobulin superfamily (IgSF) [10], the HAb18G/CD147 variant, identified through screening a hepatocellular carcinoma cDNA library with the monoclonal antibody HAb18 [57], shares an identical open reading frame with CD147, confirming its identity across tissues as a new member of the CD147 molecular family. Building on four decades of research, the developmental trajectory of CD147 can be delineated into four distinct yet interconnected phases:

Phase 1: Discovery and Identification (1980s–2000s)

The initial stage focused on defining the molecule's identity. Following the identification of TCSF [1, 31, 64] and the cloning of mouse *Basigin* [5, 31, 65, 66], research quickly established its homologs across species. Key milestones in the 1990s included mapping the chromosomal location [67], correlating expression with metastatic potential [10], and cloning HAb18G/CD147 [57, 61]. Crucially, the generation of knockout mice in this period revealed severe reproductive and neural defects [68], providing the first genetic evidence of its essential physiological roles.

Phase 2: Microenvironmental Remodeling (2000s–2010s)

The turn of the millennium marked the elucidation of CD147's proteolytic functions. Studies established its role in inducing glycosylation‐dependent matrix metalloproteinase (MMP) secretion [69], thereby promoting extracellular matrix degradation and tumor invasion. Concurrent structural breakthroughs, including the resolution of its crystal structure [70], paralleled functional discoveries such as its implication in thymocyte development [71] and β‐amyloid degradation [72]. Notably, the link to MCT1/4‐mediated lactate transport was identified [35, 73, 74], foreshadowing its metabolic significance.

Phase 3: Metabolic‐Immune Coupling (2010s–2020s)

This decade defined CD147 as a dual‐function “Energy‐Structure Coupler.” Research identified the Cyclophilin A (CyPA) binding site [75, 76] and characterized the CD147‐CyPA axis as a driver of inflammatory diseases [77, 78, 79]. Simultaneously, its role as an obligate chaperone for MCTs was solidified, linking it to chemoresistance [80, 81, 82] and metabolic reprogramming [83, 84]. The spectrum of its functions expanded to include acting as an erythrocyte receptor for *Plasmodium falciparum* [85, 86, 87] and mediating exosome‐based tumor‐stroma communication [88].

Phase 4: Clinical and Translational Advances (2020s‐Present)

Recent years have focused on cross‐disease validation and therapeutic engineering. CD147 emerged as a critical co‐receptor for SARS‐CoV‐2 [89, 90], bridging infectious disease with immunopathology [91]. Translational efforts have accelerated, with the development of exosome‐based biomarkers [13, 42, 92, 93] and demonstration of antibody efficacy in atherosclerosis [94]. Most recently, the engineering of logic‐gated CD147‐CAR‐NK cells [95] represents a leap toward precision medicine [96], aiming to overcome systemic toxicity while enhancing tumor selectivity.

This integrated timeline traces CD147's transformation from a collagenase‐inducing glycoprotein to a multifunctional therapeutic target. By encompassing oncology, immunology, and infectious disease, this evolutionary trajectory provides a robust framework for understanding cellular communication and developing precision interventions.

## 3 Structure Variants, Expression Profile, and Interaction Network

**FIGURE 2:**
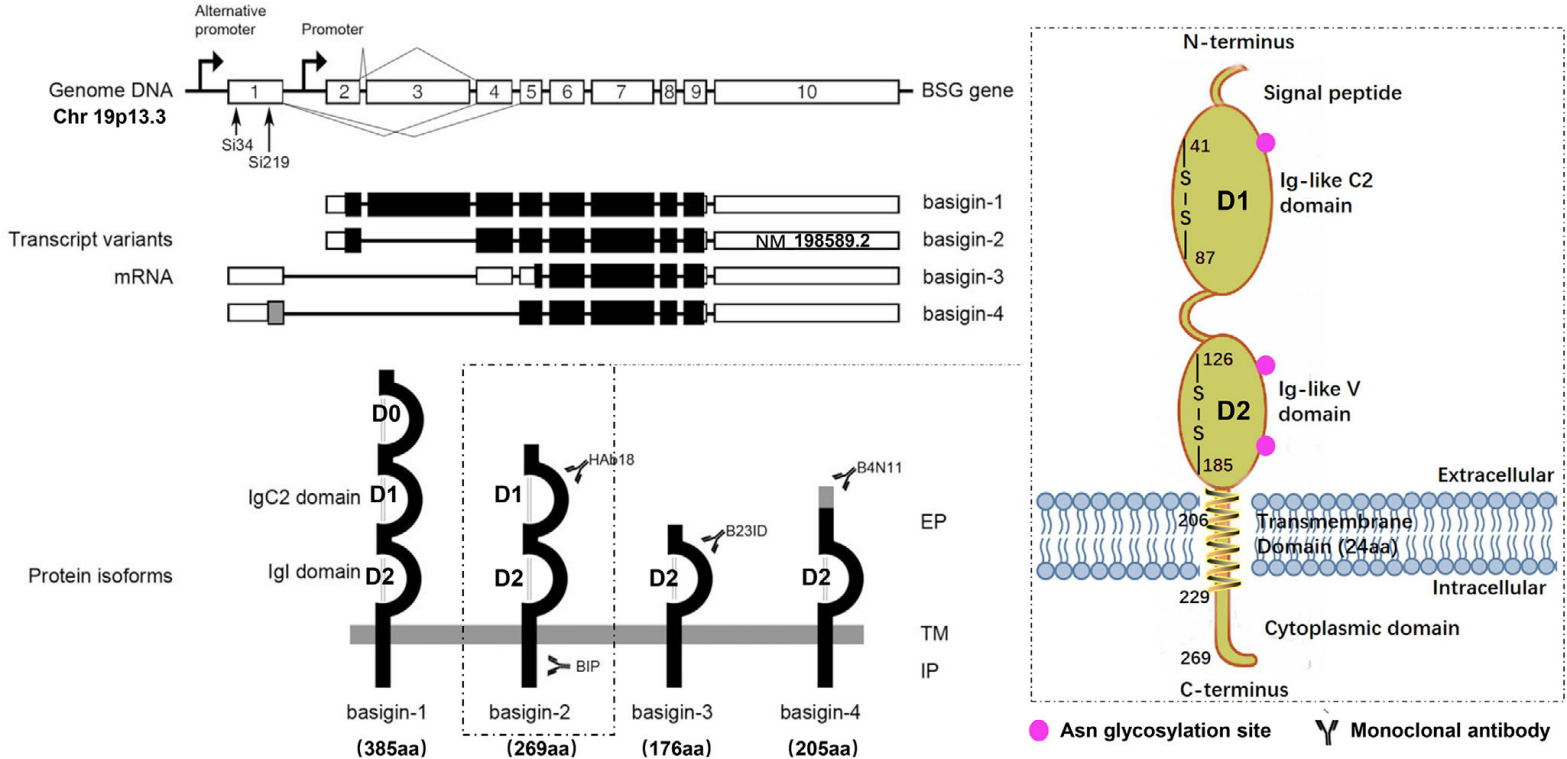
Genomic architecture, transcript variants and structural features of human CD147 (Basigin/BSG). This schematic depicts the BSG gene on chromosome 19p13.3, comprising 10 exons and alternative promoter usage that generate four distinct isoforms (Basigin‑1 to ‑4). Basigin‑2, the canonical and predominant isoform, is detailed with its domain topology: two extracellular immunoglobulin‑like domains (IgC2/IgD1 and IgI/IgD2), three N‑glycosylation sites (pink dots), a single transmembrane segment and cytoplasmic domain, with epitopes for monoclonal antibodies (e.g., B4N11, B23ID, HAb18, BIP) are indicated to highlight functional and therapeutic relevance.

This section dissects CD147's structural and regulatory intricacies, laying the groundwork for its biological functions. CD147/Basigin, a highly glycosylated, single‐pass type I transmembrane glycoprotein of the immunoglobulin superfamily (IgSF) [5, 97], is encoded by the human *BSG* (CD147) gene located on chromosome 19p13.3 [4] (Figure 2). Through alternative promoter usage and mRNA splicing, four major isoforms are generated [23]. Basigin‐2 (NM_198589) [98], encoding a 269‐amino‐acid protein with two extracellular immunoglobulin‐like domains (IgD1‐IgD2), is the predominant isoform and the most extensively studied [99]. In contrast, Basigin‐1 (NM_001728) features an additional N‐terminal IgD0 domain and is retina‐specific [100]. The biological roles of Basigin‐3 and Basigin‐4 remain less defined [23].

### 3.1 Tissue Expression Profile

CD147 exhibits a distinctive tissue‐specific pattern that varies with developmental stage and pathological context [28, 101], characterized by restricted basal expression in normal tissues versus broad overexpression in malignancy [102].

#### 3.1.1 Physiological Expression

Physiologically, CD147 is spatially restricted to metabolically active or stem‐like populations. It is predominantly found in the basal layer of epidermal keratinocytes, hair follicle roots, and activated immune cells [59, 103, 104]. Within the hematopoietic lineage, expression is constitutive but markedly upregulated upon activation in T cells, B cells, and monocytes [9, 105], whereas in thymocytes, it correlates with cellular immaturity [106]. Developmentally, CD147 expression is tightly linked to differentiation status. In human skin, it is induced by 20 weeks of gestation in progenitor cells but declines as keratinocytes undergo terminal differentiation [107, 108]. In specialized tissues, CD147 dictates functional integrity: it is essential for photoreceptor survival in the retina [74] and facilitates sperm–egg interaction [109] in the reproductive system.

Notably, in contrast to these specific niches, a comprehensive immunohistochemical survey of 239 epithelial samples revealed that CD147 is largely quiescent or weakly expressed in most normal differentiated epithelia, with an overall positivity rate of only 5.18% [102]. The majority of tissues—including the prostate, spleen, liver, and kidney—showed negligible or low (<20%) expression, with gastric tissue being a notable exception (30%) [103]. This limited physiological distribution provides a crucial safety window for therapeutic targeting.

#### 3.1.2 Pathological Overexpression

Against this background of low physiological expression, CD147 is significantly upregulated across a broad spectrum of malignancies, including hepatocellular carcinoma [102], lung cancer, breast cancer, melanoma, and lymphoma et al. [110, 111]. This aberrant overexpression serves as an independent prognostic indicator associated with poor clinical outcomes [102, 103, 104]. Functionally, high CD147 levels drive proliferation, invasion, and metabolic reprogramming [48, 84], creating a sharp contrast between malignant tissues and their normal counterparts that can be exploited for diagnostic [102, 112] and therapeutic purposes [28, 113].

### 3.2 Expression Regulation and Post‐Translational Modification

CD147 expression is tightly controlled at multi‐levels including the transcriptional, translational, and post‐translational stages according to physiological and pathological contexts.

Transcriptional Regulation: The CD147 gene features an alternative promoter region containing a TATA box and CpG islands (CGIs) [97], which are subject to epigenetic regulation through DNA methylation. This region serves as a binding platform for transcription factors, including specificity protein 1 (Sp1) [114], Sp3, early growth response protein 2 (EGR2), epithelial–mesenchymal transition (EMT) regulators (Snail and Slug), sterol carrier protein 2 (SCP‐2), and hypoxia‐inducible factor‐1α (HIF‐1α) [115]. Hypomethylation of the promoter enhances Sp1 binding, thereby upregulating CD147 transcription in tumor cells, driving its overexpression in malignancy [114].

Post‑Transcriptional and Translational Regulation: MicroRNAs (miRNAs), such as miR‐492 [116], miR‐125b‐5p, miR‐146a‐5p [117], and let‐7b [118], modulate CD147 expression by post‐transcriptional suppression or fine‐tuning. Additionally, soluble mediators—including tumor necrosis factor‐α (TNF‐α) [11], interleukins [119, 120], receptor activator of nuclear factor κB ligand (RANKL [121]), prostaglandins [122], and sex hormones (progesterone and estrogen [123])—regulate CD147 levels.

**FIGURE 3:**
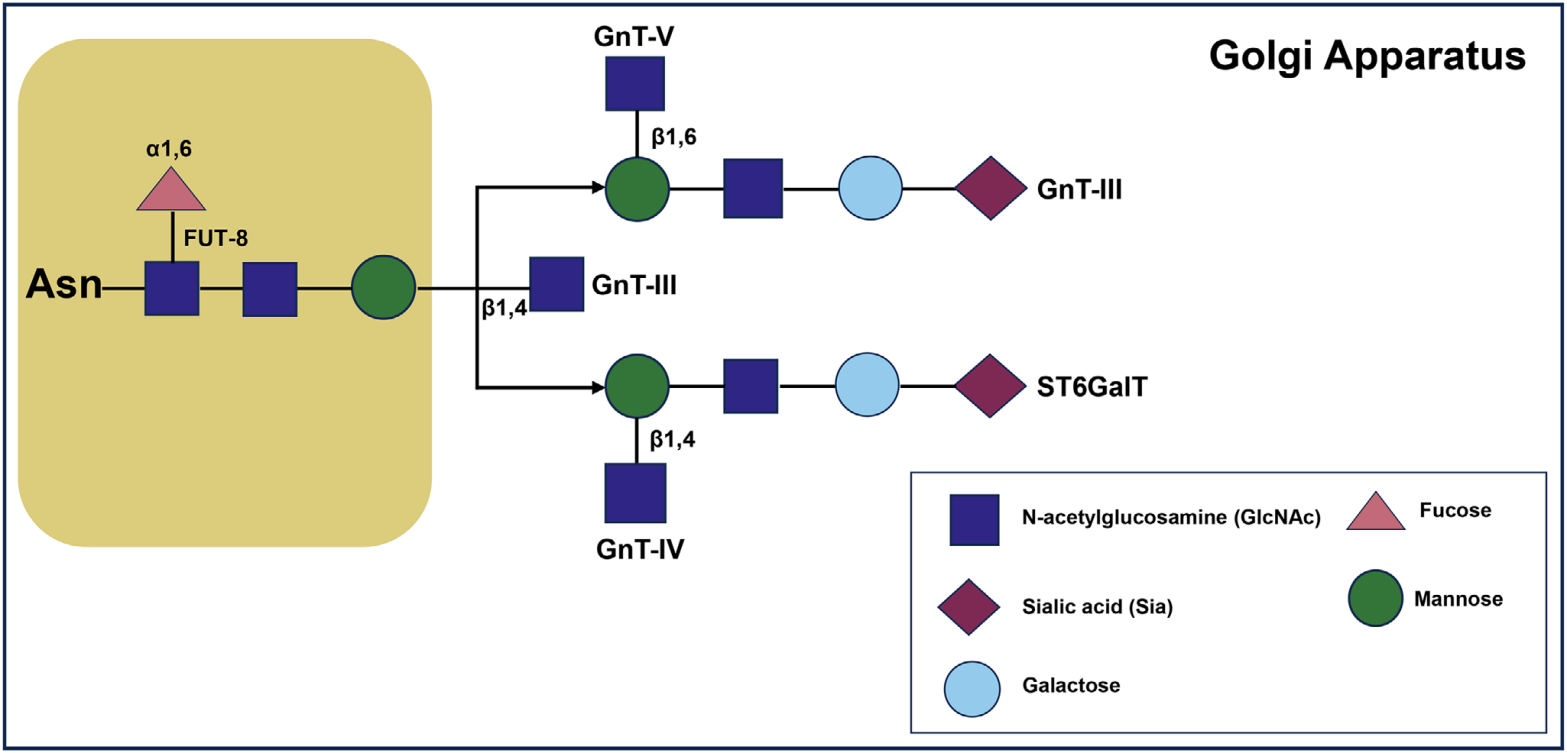
N‐glycosylation sites of CD147 and their corresponding glycosyltransferases. The diagram delineates the site‐specific N‐glycosylation modifications of CD147 at residues Asn⁴⁴, Asn^152^, and Asn^186^. In the Golgi apparatus, the core fucosylation was catalyzed by FUT8, β1,6 N‐acetylglucosamine (GlcNAc) branching by GnT‐V, β1,4 branching by GnT‐IV, and terminal sialylation by ST6GalT. Notably, the β1,6 N‐acetylglucosamine modification of the CD147 catalyzed by GnT‐V promotes hepatocellular carcinoma progression. (GnT: N‐acetylglucosamie transferase; FUT: fucosyl transferase; ST6GalT: α‐2,6 sialic acid transferase).

Post‑Translational Modifications (PTMs): Post‐translational modifications, particularly glycosylation and phosphorylation, are critical for regulating CD147's subcellular localization and functional activity [124, 125]. Phosphorylation by Fyn kinase targets tyrosine residues Y140 and Y183, modulating interactions with N‐acetylglucosaminyltransferase V (GnT‐V) and influencing subsequent glycosylation and membrane targeting [125] (Figure 3).

CD147 is predominantly N‐glycosylated [124], though O‐linked oligosaccharides are reported in specific contexts, such as chicken retinal neurons and epithelial tissues [126]. Variable N‐glycosylation produces two major glycoforms: the low‐glycosylated (LG) form, associated with non‐infectious inflammation, and the high‐glycosylated (HG) form [124], which activates matrix metalloproteinases (MMPs) to promote tumor invasion, metastasis, and extracellular matrix remodeling [69].

### 3.3 CD147 Interaction Network and Signaling Pathways

**FIGURE 4:**
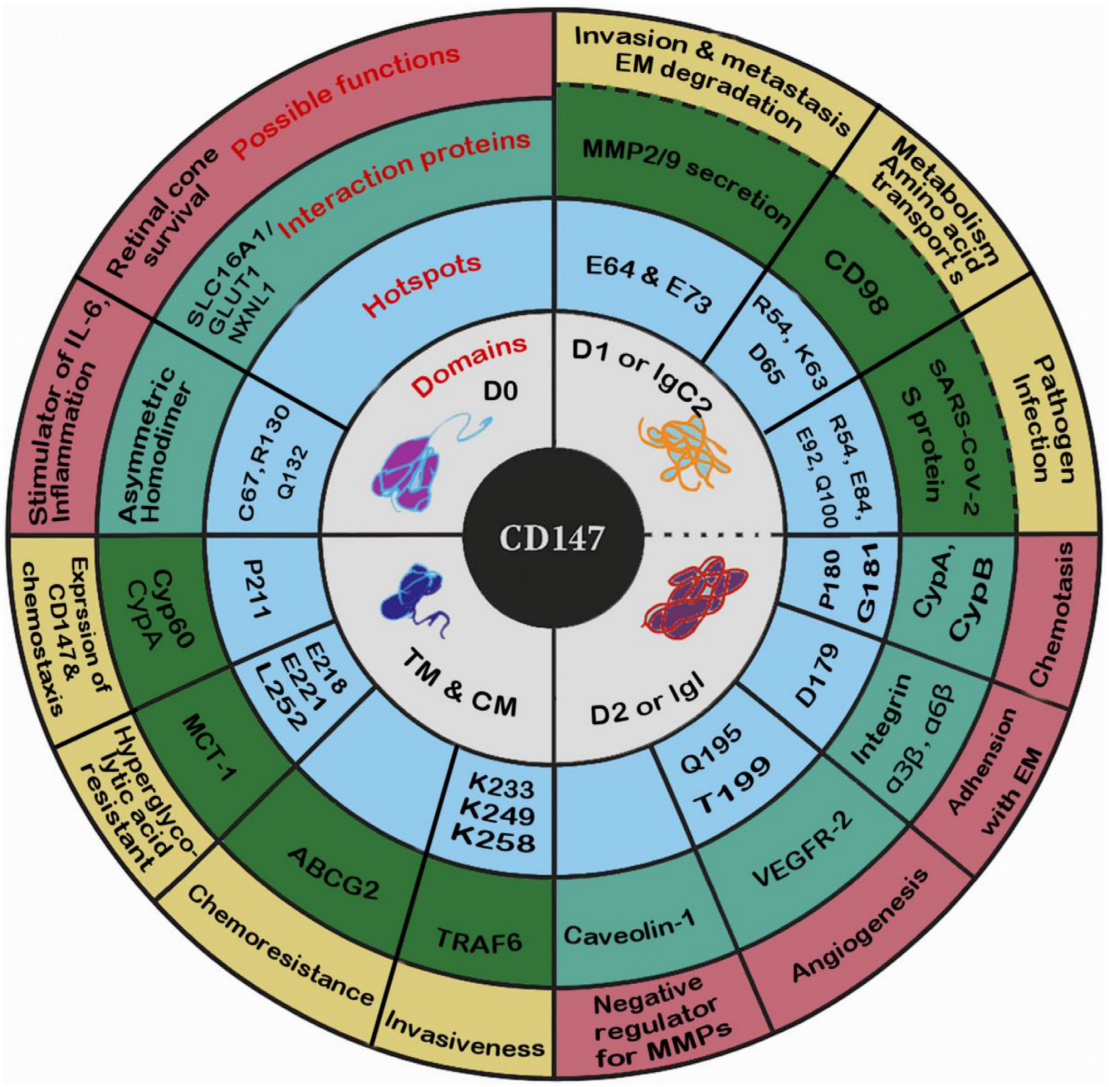
The concentric interactome of CD147 and associated binding residues. This map illustrates the multi‐layered interaction network of human CD147. The innermost ring defines the structural core (IgC2 and IgV domains). The middle concentric layer map specific binding hotspots to their respective partners, including Cyclophilins (A/B), Integrins (β1/β3), MCTs, Caveolin‐1, and VEGFR‐2. The outermost ring integrates these molecular interactions into biological outputs, ranging from chemokine induction and angiogenesis to metabolic reprogramming and pathogen invasion. Note: Novel interactors identified via high‐throughput mass spectrometry (e.g., GNAI2, RAP1B, TRAF2) are listed in the text but excluded here for visual clarity.

CD147 exerts its diverse regulatory functions through an extensive interaction network with membrane and extracellular proteins, orchestrating multiple signaling pathways that govern development, metabolism [127], immunity, and neural function. As depicted in Figure 4, CD147 engages a specific array of binding partners via distinct structural domains—primarily the extracellular IgC2 (D1) and IgI (D2) domains, as well as the transmembrane region—to coordinate these complex biological processes [128].

Specific residues within CD147's domains dictate its interactions with core partners, thereby defining its functional outputs. For instance, the L252 residue in transmembrane domain serves as a key sorting signal for the basolateral targeting of Monocarboxylate Transporters (MCT1/3/4) [129]. This interaction, which involves the IgD2 domain acting as an allosteric modulator (revealed by Cryo‐EM structures, PDB ID: 6LYY) [8, 35], is fundamental for lactate/pH homeostasis [35, 130, 131] and drives chemoresistance (via ABCG2) and the Warburg effect [8, 132]. The extracellular IgC2 (D1) domain contains critical hotspots such as Pro180/Gly181, which serve as the primary binding site for Cyclophilins (CyPA/CyPB) [133, 134]. This interaction is pivotal for chemotaxis and inflammatory signaling, and is also hijacked by pathogens such as HIV‐1 [135, 136] and SARS‐CoV‐2 (via Spike protein binding at Glu92/Gln100) [89, 90, 137]. Meanwhile, the IgI (D2) engages Integrins (α3β1 [138], α6β1 [139]) and VEGFR‐2 [140], relying on residues like Q195/T199. Affinity purification coupled with mass spectrometry has also identified additional CD147‑interacting proteins [141] such as CD45, GNAI2, CD47, Lck, RAP1B, PPP2R1B, TRAF2, VAT1 and TFF3 [142]. Other partners mapped to specific domains include CD98 [143, 144, 145] (amino acid transport) and Caveolin‐1 (MMP regulation) [146], further diversifying CD147's functional repertoire.

**FIGURE 5:**
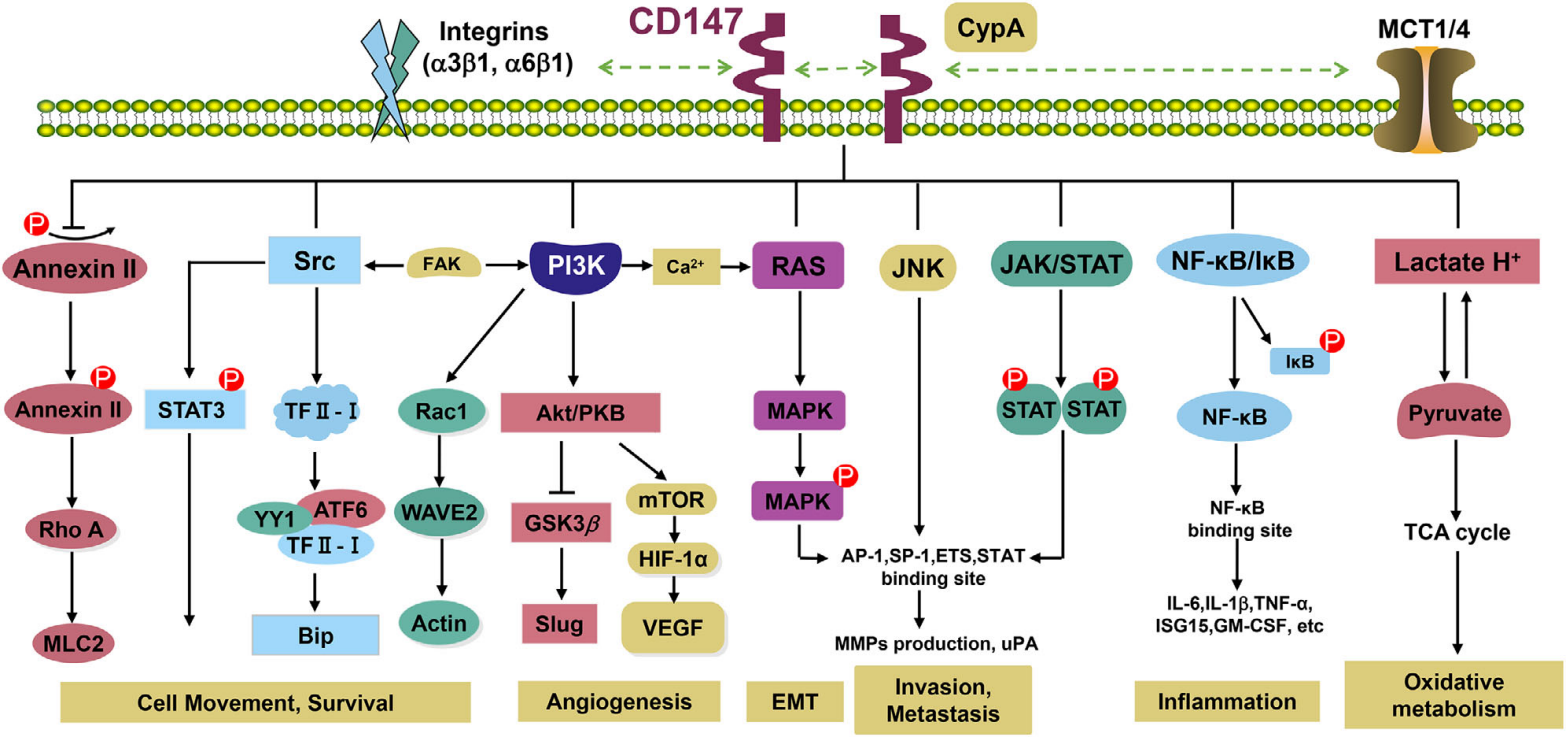
CD147 signaling transduction network and functional outputs. The schematic depicts the major signaling cascades orchestrated by CD147 via its “triad” of membrane partners: Integrins (α3β1, α6β1), Cyclophilin A (CyPA), and Monocarboxylate Transporters (MCT1/4). This complex acts as a signaling nexus, coupling lactate export (metabolism) with cytoskeletal remodeling (structure). Downstream pathways, including PI3K/Akt/mTOR, Ras/MAPK, JAK/STAT, and NF‐κB, converge to drive malignant phenotypes such as EMT, invasion, angiogenesis, and survival, effectively translating microenvironmental stimuli into gene expression changes.

These molecular interactions converge on integrated downstream signaling cascades to amplify pathological phenotypes. CD147 acts as a signaling hub, translating extracellular cues into intracellular responses primarily via the MAPK [147], PI3K/Akt [148], and NF‐κB pathways [30, 149] (Figure 5). Through the MAPK/ERK axis [150], often triggered by Integrin or CyPA binding, CD147 induces MMP production and drives epithelial‐mesenchymal transition (EMT) [151], promoting tumor invasion. In a metabolic context, CD147 enhances glycolysis via PI3K/Akt/mTOR/HIF‐1α signaling while suppressing fatty acid oxidation [38, 152]. Furthermore, CD147 modulates nuclear transcription: its intracellular domain (ICD) can translocate to the nucleus [46, 153] or activate NF‐κB (via TRAF6‐IKK‐IRF5) [94] and SMAD2/3 (via ALK5 interaction) [154, 155] to drive inflammatory and fibrotic gene expression. Collectively, these integrated networks link CD147 to metabolic transport (MCTs [8], GLUT1 [83]), immune modulation (Cyclophilins, S100A9 [149, 156, 157]), and cytoskeletal dynamics, reinforcing its role as a multifunctional regulator and high‐value therapeutic target.

## 4 Fundamental Biological Roles: The Logic of Energy‐Structure Coupling

While CD147 is frequently characterized by its pathological overexpression, its physiological distribution reveals a fundamental governing principle: CD147 is preferentially expressed in tissues exhibiting high metabolic demand coupled with continuous structural plasticity. From the rapidly dividing trophoblast to the synaptic junctions of the central nervous system, CD147 functions not merely as an adhesion molecule, but as an obligate molecular scaffold. By spatiotemporally organizing nutrient transporters (e.g., MCT1/4 [129], CD98 [143, 144]) and extracellular matrix modifiers (e.g., MMPs, integrins) into functional membrane super‐complexes, CD147 translates metabolic fuel into morphogenetic action [12, 29]. This unique “energy‐structure coupling” mechanism ensures that cells requiring rapid migration or proliferation—such as those in embryogenesis, wound healing, and retinal phototransduction—have immediate access to both the metabolic substrates (via lactate [131]/amino acid transport) and the physical path‐clearing capacity (via ECM remodeling) necessary for their function [113]. Consequently, the pathological roles of CD147 in cancer and inflammation can be viewed as a hijacking of this physiological machinery.

### 4.1 Developmental Plasticity: Orchestrating Invasion and Energy Supply

Embryonic development represents the archetype of CD147's dual function: supporting high metabolic flux while enabling tissue invasiveness. During early pregnancy, CD147 (basigin) is a critical regulator in trophoblast cells [158], where it coordinates a “tumor‐like” invasion program required for implantation through modulation of MMPs and VEGF signaling [159]. The lethality observed in knockout models confirms this metabolic‐structural dependency: Global knockout of the *Bsg* gene (*Bsg* ^−^/^−^) results in ∼70% embryonic lethality around the peri‐implantation stage. Crucially, the cause of death in surviving neonates—interstitial pneumonia—reflects a failure in lung alveolar architecture remodeling, a process heavily dependent on ECM dynamics [68]. Furthermore, the infertility seen in CD147‐deficient females is not merely a reproductive defect but a failure of the uterine tissue to undergo the necessary structural remodeling for embryo reception. Adult survivors exhibit phenotypes linked to impaired bioenergetics and signaling, including erythroid lineage defects [160], suggesting that CD147 is essential for sustaining the high‐energy demands of hematopoiesis.

### 4.2 Neural Circuitry: Coupling Lactate Shuttling with Barrier Integrity

In the central nervous system, CD147 acts as a “bioenergetic gatekeeper.” It is strategically positioned at the blood‐brain barrier (BBB) [161, 162] and neuronal synapses [163] to synchronize nutrient delivery with neural activity. In the retina, the retina‐specific isoform Basigin‐1 forms a tight complex with MCT1 and MCT4. This interaction facilitates the “lactate shuttle” required to sustain photoreceptor metabolism [100, 164]. Consequently, its deficiency leads to energy failure, resulting in rod outer segment degeneration and retinitis pigmentosa‐like phenotypes [74]. At the BBB and synapses, CD147 regulates neuron‐glia interactions and vesicle release. However, this structural role is double‐edged: while essential for plasticity, its genetic deletion alters BBB permeability via dysregulated MMP activation, leading to Alzheimer's‐like pathology and vascular leakage [163, 165, 166]. Thus, CD147 maintains neural homeostasis by balancing barrier tightness with the metabolic transport required for learning and memory [56].

### 4.3 Immune‐Metabolic Coupling: Sensing Microenvironmental Nutrient Flux

CD147 serves as a pivotal “sensor” that adapts immune cell function to nutrient availability. By integrating signals from distinct ligands, it establishes a regulatory network that dictates cell fate based on metabolic status [128].

- Lactate‐Driven Adaptation: As an obligate chaperone for MCT1/3/4, CD147 ensures their glycosylation‐dependent membrane localization. This function is critical for the “Warburg effect”: by exporting lactate [73, 131], CD147 prevents intracellular acidification while conditioning the extracellular microenvironment, a mechanism hijacked by tumors to suppress immunity [8, 73, 167].
- Amino Acid & Lipid Sensing: Beyond lactate, CD147 functions as a nutrient rheostat. By forming a super‐complex with the amino acid transporter CD98 (SLC3A2) [168], it activates the mTORC1‐SREBP1c axis to drive glutamine uptake and fatty acid synthesis [12, 152]. In macrophages, this metabolic reprogramming directly fuels pro‐inflammatory M1 polarization and arachidonic acid metabolism, positioning CD147 as a bona fide immunometabolic checkpoint rather than a simple surface marker [11, 169].

### 4.4 Tissue Repair and ECM Remodeling [29]: Balancing Fibrosis and Resolution

Wound healing recapitulates developmental programs, requiring CD147 to coordinate rapid cell migration with matrix reconstruction. CD147 stimulates fibroblasts and endothelial cells to secrete MMPs [170], thereby “clearing the path” for migration. Simultaneously, it drives myofibroblast differentiation by inducing α‐smooth muscle actin (α‐SMA) expression via the TGFβ1‐Smad2/3 axis [171]. This demonstrates a precise temporal control: CD147 first promotes the catabolic phase (MMP‐mediated degradation) to facilitate movement, and subsequently supports the anabolic phase (fibrosis/repair) [170]. Dysregulation of this balance, however, shifts the outcome from healthy repair to pathological fibrosis or chronic inflammation [172].

In summary, CD147's functional repertoire extends far beyond that of a conventional adhesion molecule. By physically coupling metabolic transporters (MCTs/CD98) with structural remodelers (MMPs/Integrins), CD147 acts as a master integrator of “Energy‐Structure” homeostasis [173]. This unifying mechanism explains its ubiquitous involvement in diverse physiological systems and highlights why its dysregulation is a common denominator in cancer, infection, and autoimmunity.

## 5 CD147 in Diseases: Hijacking the Energy‐Structure Axis

While the physiological role of CD147 is to coordinate energy metabolism and structural plasticity for development and repair, its pathological contribution represents a dysregulated “hijacking” of this machinery. Across diverse disease landscapes, CD147 drives progression through a unified pathological framework involving metabolic reprogramming, immune imbalance, and aberrant tissue remodeling [13, 15, 84]. By exploiting these fundamental cellular processes, CD147 acts as a central engine for malignancy, a gateway for pathogens, and an amplifier of chronic inflammation. This section dissects how these core mechanisms manifest in specific disease contexts.

### 5.1 CD147 in Cancer

**FIGURE 6:**
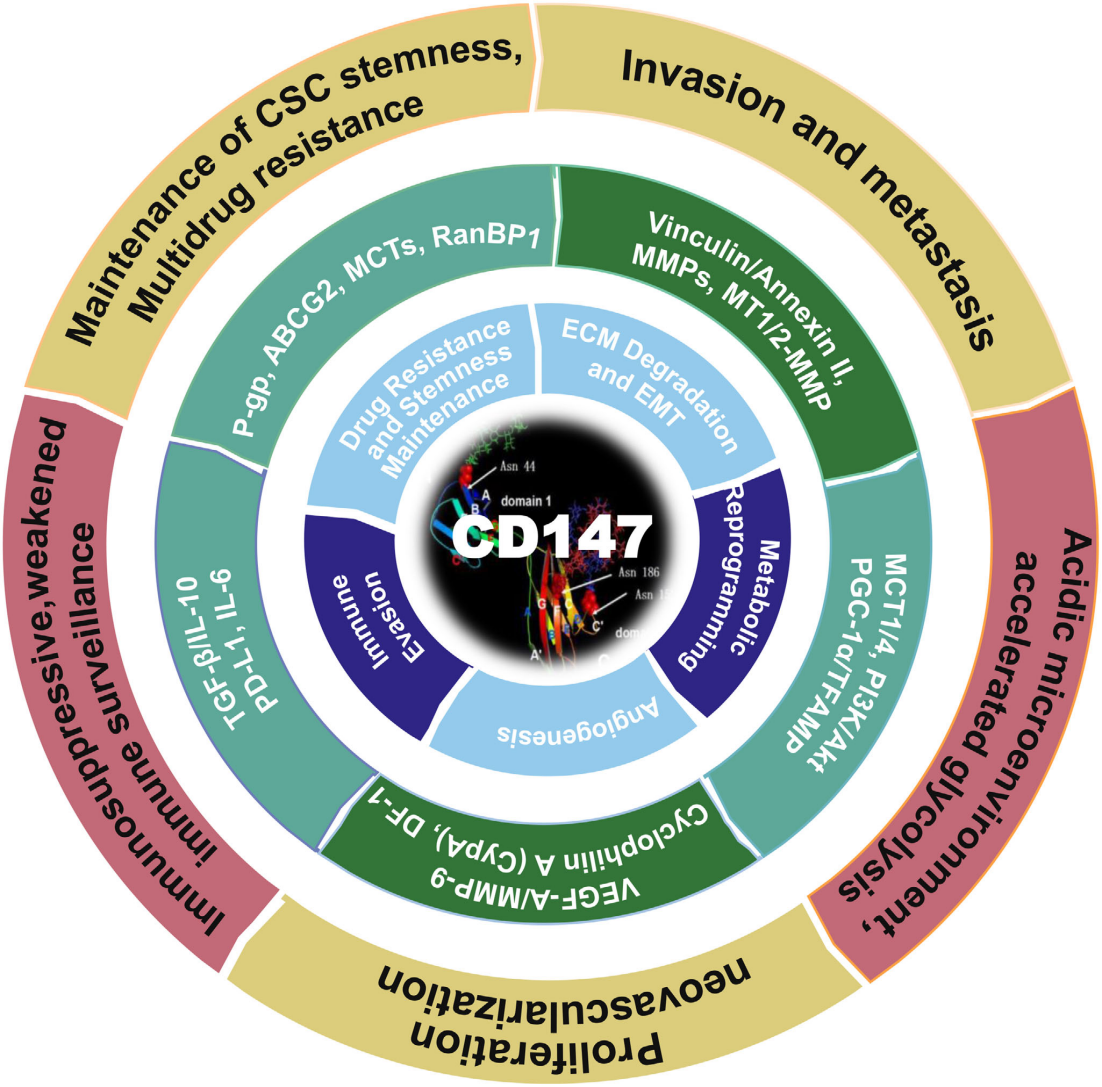
CD147 orchestrates the hallmarks of cancer progression. This diagram illustrates the central role of CD147 in the tumor ecosystem. The core highlights CD147 as a driver of malignancy. The middle ring identifies specific molecular effectors (e.g., MMPs, VEGF, TGF‐β) regulated by CD147 signaling. The outer layer maps these effectors to the distinct hallmarks of cancer, including sustained angiogenesis, tissue invasion and metastasis, immune evasion, metabolic reprogramming (Warburg effect), and drug resistance. Collectively, this highlights CD147 as a high‐value target for disrupting multiple axes of tumor survival simultaneously.

In oncology, CD147 (Basigin/EMMPRIN) is not merely a marker of poor prognosis but an active driver that confers “stem‐like” plasticity to tumor cells. By engaging a tripartite axis of invasion, metabolism, and immune evasion, it orchestrates the lethal transition from local growth to systemic metastasis (Figure 6).

#### 5.1.1 ECM Degradation and EMT

Tumor invasion relies on the physical breaching of barriers, a process directly fueled by CD147 through two synergistic mechanisms. First, CD147 initiates a paracrine amplification loop by binding vinculin or annexin II on adjacent fibroblasts. This interaction triggers the massive release of MMP‐1/2/3/11 and MT1/2‐MMPs, focusing proteolytic activity at the invasive front [174, 175, 176, 177]. Crucially, in invasive pseudopodia of breast cancer cells, the colocalization of CD147, MCT4, and MMP14 creates a “pH‐dependent invasion module,” where lactate efflux locally activates adjacent proteases, thereby accelerating ECM degradation. Second, acting through the PI3K/Akt‐ERK1/2 axis [148], CD147 upregulates transcription factors such as Snail, Slug, and Twist. This signaling cascade disrupts E‐cadherin/β‐catenin complexes to drive epithelial‐mesenchymal transition (EMT) [178, 179], effectively conferring a migratory phenotype that has been validated across colorectal, breast, and liver cancer models [180, 181, 182].

#### 5.1.2 Metabolic Reprogramming

Acting as a molecular chaperone, CD147 stabilizes MCT1/4 at the plasma membrane [131]. It establishes a positive feedback loop wherein lactate efflux prevents intracellular acidification while simultaneously creating an acidic, immunosuppressive microenvironment [8]. Mechanistically, the CD147‐MCT1 complex actively promotes p53 degradation via the PI3K/Akt‐MDM2 axis [183], effectively removing cell cycle checkpoints to enhance proliferation. Under hypoxic conditions, the CD147‐HIF‐1α‐GLUT1 axis cooperates with MAPK‐PPARα signaling to inhibit fatty acid oxidation. This shift achieves dual glucose‐lipid metabolic reprogramming, conferring a survival advantage under nutrient stress [183].

#### 5.1.3 Angiogenesis

To sustain rapid tumor growth, CD147 rewires the vascular network by synergizing with CyPA to activate HIF‐1α. This upregulates VEGF‐A and MMP‐9 while simultaneously downregulating endogenous inhibitors like thrombospondin‐1 [184, 185]. Notably, CD147 functions as a VEGFR‐2 co‐receptor [186, 187]. In fibrosarcoma models, a CD147‐IGF‐1 positive feedback loop amplifies angiogenic signaling, a pathway that can be disrupted by specific inhibitors such as forsythoside [188].

#### 5.1.4 Immune Evasion

CD147 actively excludes immune surveillance through a “Dual‐Hit” mechanism comprising both metabolic and signaling suppression. Metabolically, CD147‐driven lactate accumulation directly inhibits T‐cell proliferation and cytotoxic activity. Concurrently, CD147 signaling induces the release of TGF‐β [189] and IL‐10 to impair dendritic cell function, drives macrophage polarization toward the immunosuppressive M2 phenotype, and upregulates PD‐L1 [190]. This creates a profound “metabolic‐immune barrier” that renders tumors resistant to immune checkpoint inhibitors [38, 191].

#### 5.1.5 Drug Resistance and Stemness Maintenance

Therapeutic failure often stems from CD147‐mediated stemness and adaptation. CD147 is enriched in Cancer Stem Cells (CSCs), where it activates Wnt/β‐catenin and the unfolded protein response (UPR) signaling to sustain self‐renewal [192, 193]. It confers multidrug resistance by interacting with efflux pumps such as P‐gp and ABCG2 [80], and prevents apoptosis via the β‐TrCP/Nrf2 antioxidant pathway [101]. A critical compensatory mechanism involves the upregulation of MCT4 in response to CD147 blockade, which preserves metabolic flux in hypoxic niches and maintains metastatic potential despite therapy [194].

### 5.2 CD147 in Pathogen Infection

CD147's physiological role as a receptor makes it an unwitting “Trojan Horse” for diverse pathogens. The mechanism typically follows a two‐step sequence: first, pathogens exploit CD147 as a co‐receptor for adhesion and internalization [195]; second, binding triggers the CyPA‐CD147 axis, amplifying a host inflammatory response that often exacerbates tissue damage [196, 197].

#### 5.2.1 Viral Invasion

Current evidence suggests CD147 serves as an alternative entry route for SARS‐CoV‐2, particularly relevant in contexts where ACE2 expression is low. The viral Spike protein binds CD147, recruiting Rab5a to drive clathrin‐dependent endocytosis [90, 137, 198]—an interaction effectively inhibited by the humanized antibody Meplazumab [199]. Crucially, the subsequent “Cytokine Storm” is driven by the Spike‐CD147‐CyPA axis, which activates MAPK pathways to release IL‐6, TNF‐α, and other pro‐inflammatory mediators, mirroring the pathology of acute respiratory distress syndrome (ARDS) [197, 200]. Beyond coronaviruses, CD147 is exploited by the measles virus [76], HIV [135], and cytomegalovirus to facilitate entry or dissemination, identifying it as a broad‐spectrum host‐directed antiviral target.

#### 5.2.2 Parasite Invasion

In *Plasmodium falciparum* malaria, CD147 acts as the essential receptor for erythrocyte invasion [201]. The parasite ligand PfRH5 binds CD147, forming a complex with CyPA and RIPR. Blocking the CD147‐PfRH5 interface abrogates invasion across all parasite strains, validating it as a critical vaccine target [202]. Similarly, bacteria such as *Neisseria meningitidis* [14, 203, 204] bind CD147 to breach the blood‐brain barrier and cause meningitis [203], further reinforcing CD147's role as a universal pathogen gateway.

### 5.3 CD147 in Autoimmune Diseases

In autoimmunity [9, 205], the coupling between energy metabolism and structural remodeling becomes dysregulated: elevated metabolic flux fuels hyperactive T cells while MMP induction drives tissue destruction, creating a self‑perpetuating cycle seen in rheumatoid arthritis [205], systemic lupus erythematosus, and psoriasis [106, 206].

#### 5.3.1 The Pathological Cycle of Autoimmunity

CD147 contributes to autoimmune progression through an interconnected cycle. First, inflammatory amplification occurs when extracellular CyPA engages CD147 [207], triggering NF‐κB and MAPK cascades that release TNF‐α and IL‐6. Second, metabolic coupling acts as a critical checkpoint; for instance, in Th17 cells, CD147 complexes with GLUT1 to maximize glycolytic flux. Without this CD147‐mediated energy surge, Th17 differentiation fails, shifting the balance toward regulatory T cells (Tregs) [168, 208]. Finally, in the tissue destruction phase, CD147 on fibroblast‐like synoviocytes (FLS) induces MMP‐9 and confers resistance to apoptosis [209], leading to the hallmark pannus formation that destroys joints in RA.

#### 5.3.2 Selective Regulation of Immune Subsets

CD147 exerts selective regulatory effects on multiple immune cell subsets, acting as a “rheostat” for the immune response. In Th17 cells, CD147 interacts with GLUT1 to enhance glycolytic flux via the Akt/mTORC1 axis [210], sustaining RORγt expression and IL‐17A secretion [211]. In regulatory T cells [168], CD147 is essential for stabilizing the immunological synapse with antigen‐presenting cells; its downregulation disrupts this interaction, weakening Treg suppressive capacity and impairing peripheral tolerance [208]. Additionally, the CyPA‐CD147 axis mediates the chemotaxis of neutrophils and macrophages to inflammatory sites [207], further accelerating tissue damage.

### 5.4 CD147 in Cardiovascular Disorders

Cardiovascular pathology represents a chronic maladaptation of CD147's remodeling function. While constitutively low levels maintain vascular homeostasis, stress‐induced overexpression drives the transition from endothelial dysfunction to fibrotic failure [212, 213, 214].

#### 5.4.1 Atherosclerosis

CD147 promotes a “vulnerable plaque” phenotype through several convergent mechanisms [215]. In macrophages, it enhances the uptake of oxidized LDL [216], accelerating foam cell formation. It also impairs efferocytosis via the TRAF6–IKK–IRF5 axis [94], promoting expansion of the necrotic core. Elevated CD147 expression promotes increased MMP release that degrades the fibrous cap and raises rupture risk. Notably, part of the plaque‑stabilizing effect of statins may stem from promoting CD147 deglycosylation, which reduces its membrane stability and surface abundance [217, 218].

#### 5.4.2 Ischemia‐Reperfusion Injury

Following myocardial infarction, the CyPA‐CD147 axis triggers a burst of reactive oxygen species (ROS) via NADPH oxidase [47], expanding the zone of cardiomyocyte injury [219, 220]. In the chronic phase, glycosylated CD147 binds TGF‐β receptor I (ALK5) [154], locking cardiac fibroblasts into a collagen‐producing state. This interaction amplifies SMAD2/3 signaling and upregulates CTGF, identifying CD147 as a key gatekeeper of cardiac fibrosis and heart failure progression [154].

### 5.5 CD147 in Metabolic Diseases

CD147 serves as a critical molecular hub linking nutrient sensing to systemic metabolic dysregulation, including obesity, type 2 diabetes (T2DM) [221], and non‐alcoholic fatty liver disease (NAFLD) [222, 223].

#### 5.5.1 Obesity and Insulin Resistance

In adipose tissue, CD147 drives a state of “meta‐inflammation” [224]. It establishes a paracrine loop with macrophage‐derived CyPA to promote M1 macrophage infiltration and, via the Akt/mTORC1‐SREBP1c axis, drives fatty acid synthesis while inhibiting lipolysis [152]. Furthermore, CD147 interferes with insulin signaling through two complementary mechanisms: it inhibits the AMPK‐SIRT1 axis, reducing GLUT4 membrane translocation; and it blocks SLC16A11‐mediated lactate efflux, causing intracellular lactate accumulation that inhibits IRS‐1 signaling [124]. These actions collectively exacerbate systemic insulin resistance [225].

#### 5.5.2 Hepatic Lipid Metabolism

In the liver, CD147 promotes steatosis by upregulating the SREBP‐1c pathway for lipid synthesis and suppressing CPT1A‐mediated fatty acid β‐oxidation [152, 226]. Liver‐specific knockout of CD147 has been shown to attenuate steatosis and inflammation, positioning it as a therapeutic target for halting the progression from NAFLD to NASH [227].

### 5.6 Others

The widespread expression of CD147 reflects a conserved role in governing cell‑state transitions across physiological systems. In the central nervous system, CD147 plays a detrimental role in Alzheimer's disease pathology [228]. It acts as a competitive inhibitor of the low‐density lipoprotein receptor‐related protein 1 (LRP1 [229]) for Amyloid‐β (Aβ) binding, thereby impeding Aβ clearance across the blood‐brain barrier and trapping it within the brain parenchyma [72, 230, 231]. Additionally, CD147 has been identified as a regulatory subunit of γ‐secretase [230], potentially increasing Aβ production. Emerging evidence links CD147 to mitochondrial ANT1 [232], where it contributes to the regulation of oxidative phosphorylation. In macrophages, CD147 deficiency reduces ROS production, implicating the molecule in inflammaging, the chronic low‑grade inflammation that accompanies aging [94, 216].

In summary, whether driving the Warburg effect in tumors, facilitating parasite entry in malaria, or amplifying the metabolic program of Th17 cells, CD147 consistently acts as a multiplier of pathology by dysregulating the energy–structure axis. This convergent mechanism suggests that therapeutic strategies developed in oncology, such as targeting CD147‐MCT complexes, may be repurposed for metabolic and autoimmune diseases, offering a unified, precision medicine approach across distinct disease contexts.

## 6 Clinical Application of CD147: From Bench to Bedside

As of August 2025, a total of 29 interventional or observational clinical trials targeting CD147 (Basigin/BSG) have been registered on ClinicalTrials.gov, spanning five major therapeutic areas: oncology, infectious diseases, autoimmune/inflammatory disorders, cardiovascular diseases, and ophthalmology (Table 1). Cumulative evidence from completed studies indicates an acceptable safety profile and encouraging clinical efficacy across multiple disease contexts, although several early‑phase programs were discontinued prior to enrollment or due to strategic decisions, highlighting the challenges of translating CD147‑targeted approaches into late‑stage development.

**TABLE 1:** Clinical trials targeting CD147 across major disease areas (Up to August 2025).

Category	NCT number	Study title	Phase	Conditions	Interventions	Study status	Key outcomes/notes	Reference
**Oncology** (8 trials)	NCT04045847	CD147‐CART cells in patients with recurrent malignant glioma	Phase 1	Recurrent glioblastoma, CD147 positive	Biological: CD147‐CART	Completed	ORR 40%, mPFS 4.2 months; favorable safety in animal models with >70% tumor inhibition.	[237]
	NCT03993743	CD147‐targeted CAR‐T by hepatic artery infusions for advanced HCC	Phase 1	Advanced hepatocellular carcinoma	Biological: CD147‐CART	Completed	DCR 58%, mOS 11.3 months; no severe toxicities, prolonged survival trend.	[237]
	NCT04841421	^89^Zrlabeled antiCD147 monoclonal antibody PET imaging	Phase 1/2	Solid tumors	Drug: 89Zr‐CD147	Unknown	Tumor uptake SUVmax 8.4; interventional imaging optimization.	[238]
	NCT05013372	CD147‐CAR T for relapsed/refractory T‐NHL	Phase 1	T‐cell non‐Hodgkin's lymphoma	Drug: CD147‐CART cells	Completed	CR 33%, neurotoxicity 12%; interventional.	[237]
	NCT06720298	CD147 peptide probe for PET imaging	Early Phase 1	Solid tumors	Drug: 18F‐FDG probe	Not yet recruiting	Imaging optimization for CD147 expression detection.	[238]
	NCT06646952	CD147 nanobody probe for PET imaging	Early Phase 1	Solid tumors	Drug: 18F‐FDG probe	Recruiting	Nanobody development for noninvasive CD147 tumor detection.	[238]
	NCT00829465	Licartin (^131^I‐CD147) + TACE for HCC	Phase 3	Hepatocellular carcinoma	Drug: Licartin	Completed	Prolonged median overall survival (26.7 vs 20.6 months); reduced recurrence.	[233, 236]
	NCT05574166	SP‐8356 (CD147 inhibitor) for cancer	Phase 1	Solid tumors	Drug: SP‐8356	Terminated	Interventional; Terminated due to strategic decision, no efficacy/safety data reported	[247, 259, 260]
**Infectious Diseases** (8 trials)s	NCT04275245	Meplazumab for COVID‐19 pneumonia	Phase 2/3	COVID‐19	Drug: Meplazumab	Completed	Interventional; improved recovery and viral clearance.	[199, 239, 240]
	NCT05813587	Meplazumab for post‐COVID	Phase 3	Post‐COVID	Drug: Meplazumab	Completed	Symptom relief 68% in post‐COVID symptoms	[199, 239, 240]
	NCT05679479	Meplazumab for severe COVID‐19	Phase 3	COVID‐19	Drug: Meplazumab	Completed	Viral clearance 3d vs 13d; reduced mortality (P<0.05), increased discharge without oxygen (82.9% vs 70.7%, P = 0.0337).	[199, 239, 240]
	NCT05679492	Meplazumab for COVID‐19	Phase 3	COVID‐19	Drug: Meplazumab	Withdrawn	Withdrawn prior to enrollment	—
	NCT05113784	Meplazumab in COVID‐19	Phase 2/3	COVID‐19	Drug: Meplazumab	Completed	Add‐on therapy; favorable safety (<5% drug‐related AEs).	[199, 239, 240]
	NCT04586153	Meplazumab for COVID‐19	Phase 2/3	COVID‐19	Drug: Meplazumab	Completed	Enhanced prognosis and viral load reduction.	[199, 239, 240]
	NCT04327310	Meplazumab (malaria)	Phase 1	Malaria	N/A	Withdrawn	—	—
	NCT06040346	Meplazumab for malignant malaria	Phase 2	Malaria	Drug: Meplazumab	Recruiting	Blocks CD147‐RAP2 interaction; FDA orphan drug designation.	[241]
**Autoimmune/** **Inflammatory Disorders** (4 trials)	NCT06287125	Renal assessment in SSc/SLE patients	NA	Systemic Sclerosis, Systemic Lupus Erythematosus	Device: Renal MRI/US, Serum CD147	Not yet recruiting	CD147 correlation with eGFR; observational.	—
	NCT02760914	CD147 in Coronary Heart Disease	NA	Coronary heart disease	N/A	Active, not recruiting	Observational; CD147 adipose expression.	—
	NCT04430764	Evaluation of EMMPRIN expression in gingival crevicular fluid (GCF) in periodontitis	NA	Periodontitis	Procedure: Non‐surgical treatment	Completed	GCF CD147 decreased 35% post‐Tx; interventional.	[243]
	NCT00035880	ABX‐CBL for acute GVHD	Phase 2/3	Acute GVHD	Drug: ABX‐CBL	Terminated	No improvement over ATG; 100‐day survival 52% vs 22% in low dose.	[242]
**Cardiovascular Diseases** (2 trials)	NCT06572267	Meplazumab for coronary artery disease	Phase 2	Coronary artery disease	Drug: Meplazumab	Recruiting	Ongoing; primary endpoint: change in plaque volume and stability	[94]
	NCT02760914	Adipose tissue in CHD	NA	Coronary heart disease	N/A	Active, not recruiting	Observational; CD147 expression in adipose.	—
**Other Areas** (7 trials)	NCT06118203	Semaglutide in pulmonary embolism	NA	Pulmonary embolism	Drug: Semaglutide	Completed	Indirectly related to CD147 via vascular inflammation pathways; no direct CD147 targeting	—
	NCT04805359	Hypoxia on erythrocyte CD147	NA	Hypoxia	Behavioral: Hypoxic exercise	Completed	Exploratory study; changes in erythrocyte CD147 expression under hypoxic conditions	—
	NCT00757120	Biomarkers and genetic factors related to emphysema	NA	COPD/Emphysema	N/A	Completed	Serum CD147 increased with severity; observational.	—
	NCT03358979	EMMPRIN in dry eye syndrome	NA	Dry eye	N/A	Recruiting	Observational; 3D distribution of EMMPRIN.	[244]
	NCT02891655	EMMPRIN in Keratoconus	NA	Keratoconus	Other: Immunohistochemistry	Completed	CD147 increased in corneal stroma; observational.	—
	NCT02891694	EMMPRIN in corneal erosion	NA	Corneal erosion	Other: Immunohistochemistry	Completed	CD147‐MMP9 correlation; observational.	—
	NCT03990740	EMMPRIN in Keratoconus	NA	Keratoconus	Procedure: Corneal sampling	Withdrawn	Observational; Withdrawn prior to initiation	—

This table summarizes 29 interventional or observational clinical trials involving CD147 (Basigin/EMMPRIN) registered on ClinicalTrials.gov up to August 2025. Trials span oncology (e.g., Licartin radioimmunotherapy for HCC, CAR‐T therapies), infectious diseases (e.g., Meplazumab for COVID‐19 and malaria), autoimmune/inflammatory disorders (e.g., ABX‐CBL for GVHD), cardiovascular diseases, and ophthalmology/other indications (e.g., ophthalmic and metabolic conditions). For each study, the NCT identifier, intervention, trial phase, recruitment status, and key reported outcomes are summarized. Abbreviations: ORR, objective response rate; mPFS, median progression‑free survival; DCR, disease control rate; mOS, median overall survival; CR, complete response; GCF, gingival crevicular fluid; Tx, treatment; NA, not applicable.

### 6.1 Oncology

The most mature clinical evidence for CD147‑targeted therapy is in hepatocellular carcinoma. Iodine (^131^I) metuximab injection (Licartin) [233], a novel ^131^I‐labeled HAb18G/CD147‐specific monoclonal antibody F(ab′)_2_ fragment, was the first radioimmunotherapy agent targeting CD147 approved by the Chinese State Food and Drug Administration in 2005 for unresectable primary liver cancer. Clinical studies have demonstrated its anti‑recurrence efficacy: in post‑liver transplantation for HCC, Licartin reduced recurrence rates by 30.42% and increased survival by 20.62% [233]; following radiofrequency ablation, it reduced one‑year recurrence by 43.5% and 2‑year recurrence by 17.5% [234]. In CD147‑positive HCC, adjuvant administration after hepatectomy improved survival, lowered early recurrence risk, and was well tolerated [235]. A multicenter Phase IV trial combining Licartin with TACE for unresectable HCC confirmed selective targeting of CD147‑overexpressing tumor cells, delayed recurrence, and prolonged overall survival [236].

Chimeric antigen receptor (CAR)‑T cell therapy targeting CD147 is under active investigation. Multiple registered trials (e.g., NCT05013372, NCT04045847, NCT03993743) are evaluating CD147‑CAR‑T constructs incorporating an anti‑human CD147 scFv–CD8–4‑1BB–CD3ζ design [237]. Clinical programs have been initiated for HCC (Bashepatocel, Basigin CAR‑TH), glioblastoma (Basgliomacel, Basigin CAR‑TG), and T‑cell lymphoma (Baslymomacel, Basigin CAR‑TL). Early data indicate favorable safety with no dose‑limiting toxicities, supporting their potential as novel cancer immunotherapeutics.

In diagnostics and imaging, the HAb18G/CD147 immunohistochemistry kit (Cametin) [102], launched in China in 2013, enables histopathological detection of multiple malignancies. Analysis of 1800 tissue samples showed low CD147 expression in embryonic and normal tissues, with an overall tumor positivity rate of 83.00% and an average positivity rate of 82.67%; cross‑reactivity in control tissues was low (5.10% overall; 5.05% average) [102]. CD147‑targeted PET imaging probes (NCT06646952, NCT06720298, NCT04841421) are in early‑phase clinical development for precision molecular imaging of solid tumors [238].

### 6.2 Infectious Disease

The COVID‑19 pandemic accelerated repurposing efforts. Meplazumab, a humanized anti‑CD147 IgG2, demonstrated reductions in viral load and mortality in severe SARS‑CoV‑2 infection in randomized studies, including an international multicenter Phase II/III study across 15 sites (NCT04275245, NCT04586153, NCT05679479) and a multicenter Phase III study in China [199, 239, 240]. Compared with placebo, Meplazumab significantly reduced all‑cause mortality within 56 days (*p* < 0.05), increased the proportion of patients discharged without oxygen support by day 28 (82.9% vs. 70.7%, *p* = 0.0337), improved prognosis, reduced viral load, and accelerated viral clearance. Across Phase I–III studies, safety and tolerability were favorable, with drug‑related adverse events occurring in <5% of patients, which were mostly mild to moderate in severity; only two infusion‑related reactions were reported. In malignant malaria, Meplazumab blocks the CD147‐RAP2 interaction, preventing *Plasmodium falciparum* merozoite invasion of erythrocytes (NCT06040346) [241].

### 6.3 Autoimmune Disease

ABX‑CBL, an anti‑CD147 monoclonal antibody (NCT00035880), was evaluated for acute graft‑versus‑host disease (GVHD) [242]. Early Phase II results showed a 100‑day survival rate of 52% in the 0.1–0.3 mg/kg group versus 22% in the 0.01 mg/kg control. Development was discontinued due to high immunogenicity (murine origin), high production costs, and limited therapeutic advantage, underscoring challenges in early‑stage monoclonal antibody development, including target validation, indication selection, and commercialization. Other exploratory studies, such as NCT04430764, have examined EMMPRIN expression in gingival crevicular fluid in periodontitis [243], revealing post‑treatment reductions of approximately 35%, suggesting a potential role for CD147 in periodontal inflammation.

### 6.4 Cardiovascular and Ophthalmic Research

Multiple studies have examined CD147 in corneal diseases such as keratoconus (NCT02891655, NCT03990740) and recurrent corneal erosion syndrome (NCT02891694), implicating it in corneal matrix remodeling via MMP regulation. NCT03358979 investigated the 3D distribution of EMMPRIN on conjunctival epithelial cells in severe dry eye [244], providing a mechanistic basis for topical anti‑CD147 therapies. In cardiovascular disease, NCT06572267 assesses Meplazumab in coronary artery disease, potentially via modulation of vascular inflammation and plaque stability.

**FIGURE 7:**
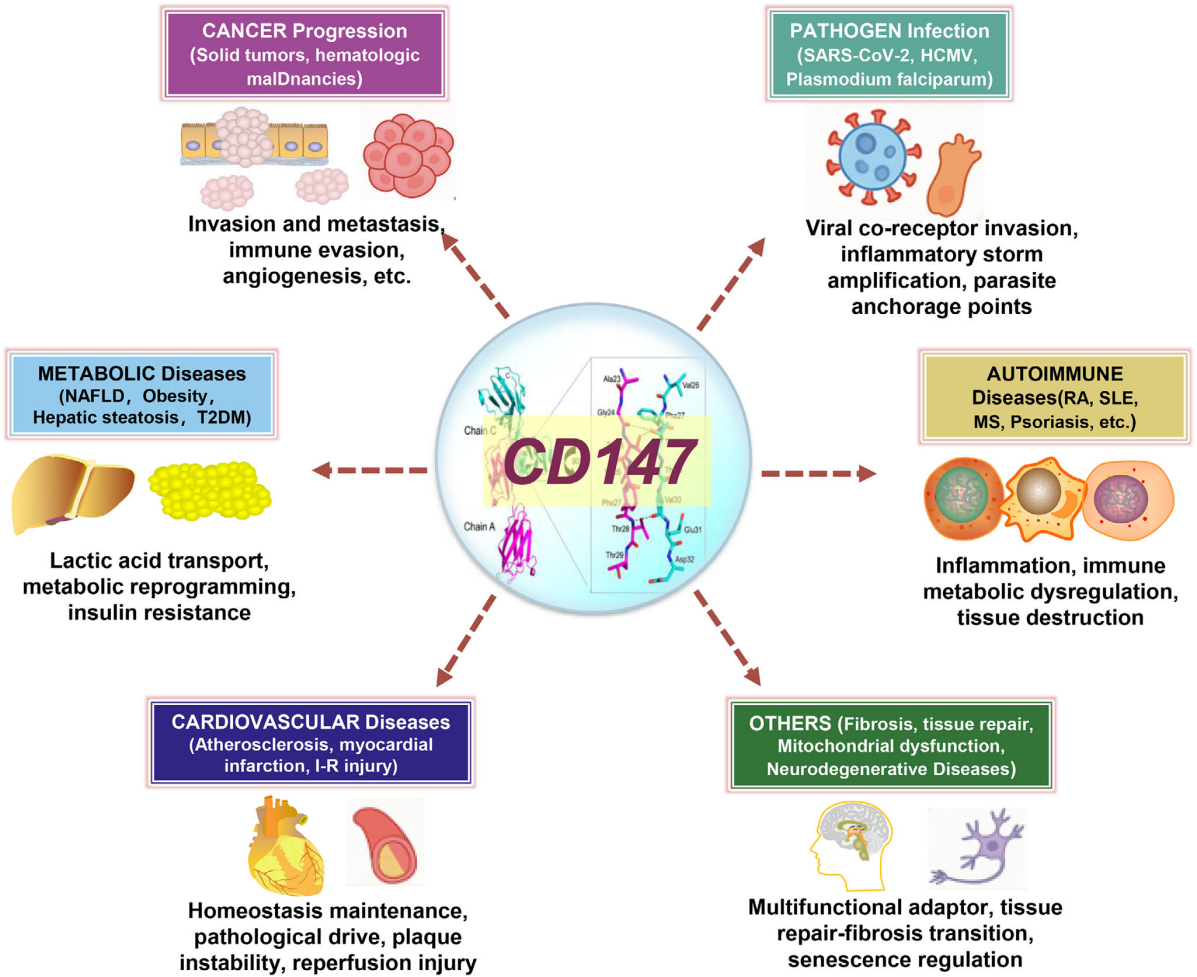
The pleiotropic landscape of CD147 across diverse pathological conditions. An integrative schematic showcasing the “Hub” function of CD147 (Basigin/BSG) beyond oncology. The figure categorizes its involvement into four major pathological domains: (1) Pathogen Infection (serving as a receptor for SARS‐CoV‐2, Malaria, etc.); (2) Cardiovascular Disease (driving plaque instability and ischemia‐reperfusion injury); (3) Metabolic Disorders (linking to insulin resistance and hepatic steatosis); and (4) Autoimmune/Neurodegenerative conditions. This underscores the molecule's role as a universal pathological adaptor across diverse tissue environments.

Overall, CD147 clinical development has entered a new phase, advancing in parallel across multiple fields (Figure 7). Approaches include monoclonal antibodies, CAR‑T cells, and imaging probes, targeting diseases from oncology and infection to autoimmunity, cardiovascular disorders, and ophthalmic disorders.

## 7 Barriers to Clinical Translation: Lessons from Success and Failure

Despite extensive preclinical validation and the encouraging efficacy of agents like Licartin and Meplazumab, the progression of CD147‐targeted interventions into broad clinical practice faces critical hurdles. These include siRNA [245], small‑molecule inhibitors [246, 247] (e.g., AC‑73 [248], PAB [249]), monoclonal antibodies [172, 250] (e.g., HAb18, M6), and immune cell products [95, 251] (e.g., CD147‑CART [237]) —each of which requires careful optimization to overcome translational barriers. An analysis of discontinued or terminated trials (Table 2) reveals that failure often stems not from a lack of target relevance, but from challenges in specificity, pharmacokinetics, and complex biology. Here, we dissect the underlying causes of these clinical setbacks and propose actionable solutions to overcome them.

**TABLE 2:** Analysis of Terminated, Withdrawn, or Discontinued Clinical Trials Targeting CD147.

NCT number	Intervention (Drug/Agent)	Indication	Phase	Status	Potential causes for termination/withdrawal	Proposed future solutions
NCT00035880	ABX‐CBL (Murine anti‐CD147 IgM)	Acute graft‐versus‐host disease (GVHD)	Phase 2/3	Terminated	Lack of efficacy: No significant survival benefit over standard therapy (ATG). Immunogenicity: Murine origin likely induced HAMA (Human Anti‐Mouse Antibody) response. Dosing limits: Potential “antigen sink” effect by erythrocytes.	Use Humanized or Fully Human antibodies (e.g., humanized Meplazumab). Explore Bispecifics (e.g., CD147 x CD3) to enhance T‐cell engagement rather than simple blockade.
NCT05679492	Meplazumab	COVID‐19	Phase 3	Withdrawn	Feasibility: Withdrawn prior to enrollment, likely due to shifting pandemic dynamics and declining case numbers. No safety signal: Not withdrawn due to toxicity.	Shift focus to pan‐viral indications (e.g., broad‐spectrum antiviral trials). Maintain “shelf‐ready” protocols for future outbreaks.
NCT04327310	Meplazumab	Malaria	Phase 1	Withdrawn	Operational: Logistics or funding challenges in early‐phase setup. Strategy change: Shifted focus to other indications or updated protocol (see active NCT06040346).	Establish stable international collaborations for tropical disease trials. Utilize vaccine strategies targeting the CD147‐PfRH5 interface as an alternative to mAbs.
NCT03990740	Diagnostic (Corneal sampling)	Keratoconus	N/A (Obs)	Withdrawn	Recruitment failure: Study withdrawn prior to initiation, likely due to difficulty in obtaining invasive corneal samples	Develop non‐invasive imaging (e.g., in vivo confocal microscopy) or tear fluid assays (sCD147) instead of tissue biopsy
NCT05574166	SP‐8356 (Synthetic small molecule)	Advanced solid tumors	Phase 1	Terminated	Strategic decision: Sponsor decision; specific data not disclosed. Potential bioavailability: Small molecules targeting protein‐protein interactions (PPIs) often face stability or potency challenges in vivo	Utilize Cryo‐EM guided structure‐based design to improve affinity. Explore PROTAC technology to degrade CD147 rather than just inhibiting the interaction.

*Note*: HAMA: Human Anti‐Mouse Antibody; ATG: Anti‐Thymocyte Globulin; PPI: Protein‐Protein Interaction. Analysis based on ClinicalTrials.gov records and associated literatures.

### 7.1 The Specificity Paradox

A primary barrier to the widespread clinical application of CD147‐targeted therapies is the molecule's broad physiological distribution, which creates a narrow therapeutic window for systemic interventions. CD147 is constitutively expressed on diverse healthy tissues, including the renal epithelium, retina, and crucially, erythrocytes (Ok blood group [59]), creating a formidable “antigen sink.” For systemically administered agents, this widespread expression can lead to rapid sequestration by red blood cells, resulting in poor tumor accumulation and potential hematological toxicities such as anemia. This limitation was evident in early trials of the murine antibody ABX‐CBL (NCT00035880) for GVHD [242], which were terminated partly due to dosing constraints and a lack of therapeutic advantage imposed by this “sink effect.” In contrast, the clinical success of Licartin (^131^I‐metuximab) in hepatocellular carcinoma highlights the importance of delivery strategy. Licartin's efficacy stems largely from its administration via hepatic artery infusion (TACE) [252], which achieves high local concentrations to leverage the radionuclide's cytotoxic power against tumor cells while minimizing systemic exposure and off‐target binding. Therefore, for future systemic therapies to replicate this success, they must bypass the “antigen sink” through logic‐gated delivery strategies. Approaches such as pro‐antibodies—which remain masked until activated by tumor‐specific proteases—or pH‐sensitive binders that engage only in the acidic tumor microenvironment are promising. Additionally, targeting tumor‐associated glycoforms (e.g., β1,6‐GlcNAc‐branched CD147) rather than the protein core offers a promising route to selectively target malignant cells while sparing healthy erythrocytes.

### 7.2 Overcoming Biological Complexity: Immunogenicity, Resistance, and Mechanism

Beyond the challenge of tissue specificity, clinical translation is further impeded by the inherent biological complexity of CD147, encompassing issues of immunogenicity, pathway redundancy, and mechanistic uncertainty. Early‐generation agents such as ABX‐CBL underscored the risk of immunogenicity; its murine origin likely contributed to trial termination, necessitating a shift toward fully humanized antibodies (e.g., Meplazumab) or nanobody‑based formats for future development. However, even with reduced immunogenicity, monotherapy often fails due to pathway redundancy, where blocking CD147 triggers compensatory upregulation of alternative adhesion molecules or metabolic transporters (e.g., MCT2), thereby fueling resistance. To counter this, bispecific antibodies (e.g., CD147×PD‐L1 or CD147×CD3) represent a rational strategy to engage multiple synergistic mechanisms simultaneously, turning a “cold” target into a potent immunotherapeutic anchor. Compounding these challenges is the rapid turnover and shedding of surface CD147 into soluble forms (sCD147), which can act as “decoys” to neutralize therapeutic antibodies. Addressing this pharmacokinetic hurdle requires novel modalities: proteolysis‑targeting chimeras (PROTACs) to catalyze intracellular degradation, or antibody‑drug conjugates (ADCs) exploiting CD147's high internalization rate to deliver cytotoxic payloads. Finally, a fundamental “driver vs. passenger” controversy remains regarding whether therapeutic benefit derives from blocking extracellular MMP induction or disrupting transmembrane metabolic transport. Since current antibodies primarily target the extracellular domain, potentially leaving the intracellular “Warburg engine” intact, structure‑guided design of small molecules disrupting the CD147‑MCT1 transmembrane interface is urgently needed to precisely cripple metabolism‑driven malignancies.

Therefore, to advance CD147 translational research, accelerated efforts must focus on overcoming barriers of specificity, immunogenicity, and resistance. Bridging this gap requires a decisive shift from simple “blocking” antibodies to next‑generation modalities—conditionally activated binders, PROTACs, and bispecifics—that respect the molecule's complex biology while maximizing therapeutic index.

## 8 Research Priorities: A Roadmap for Translational Success

Addressing the translational gap requires a strategic shift from broad blockade to precision modulation, starting with the elucidation of CD147's structural duality. Current strategies often ignore the protein's conformational plasticity. Future efforts must leverage advanced structural biology (e.g., cryo‐EM [253]) to visualize the spatiotemporal dynamics that allow CD147 to switch between its “metabolic chaperone” and “invasion promoter” states. Resolving these transitions is the prerequisite for designing allosteric inhibitors that selectively disrupt specific pathogenic interfaces (e.g., CD147–MCT1) while preserving physiological homeostasis.

Concurrently, overcoming systemic toxicity necessitates the development of microenvironment‐responsive, “logic‐gated” therapeutic platforms [51]. To circumvent the erythrocyte antigen sink, delivery systems must exploit pathological cues as triggers—such as pro‐antibodies masked by protease‐cleavable peptides or pH‐sensitive binders active only in the acidic tumor milieu [254]. Furthermore, discriminating tumor‐associated glycoforms [42] (e.g., β1,6‑GlcNAc‐branched glycans) from physiological variants offers a precise chemical strategy to enhance selectivity, directly addressing the “specificity paradox” limiting current trials [255].

Optimizing clinical outcomes also necessitates dissecting genetic heterogeneity and resistance evolution. Integrating single‐cell multi‐omics with longitudinal sampling is essential to track clonal dynamics, particularly how functional polymorphisms (e.g., rs8259 [256, 257]) influence drug efficacy. Moreover, distinguishing whether resistance arises from antigen loss or metabolic reprogramming (e.g., a shift to oxidative phosphorylation) will guide rational combination strategies, such as pairing CD147 blockade with mitochondrial inhibitors to mitigate adaptive escape.

**FIGURE 8:**
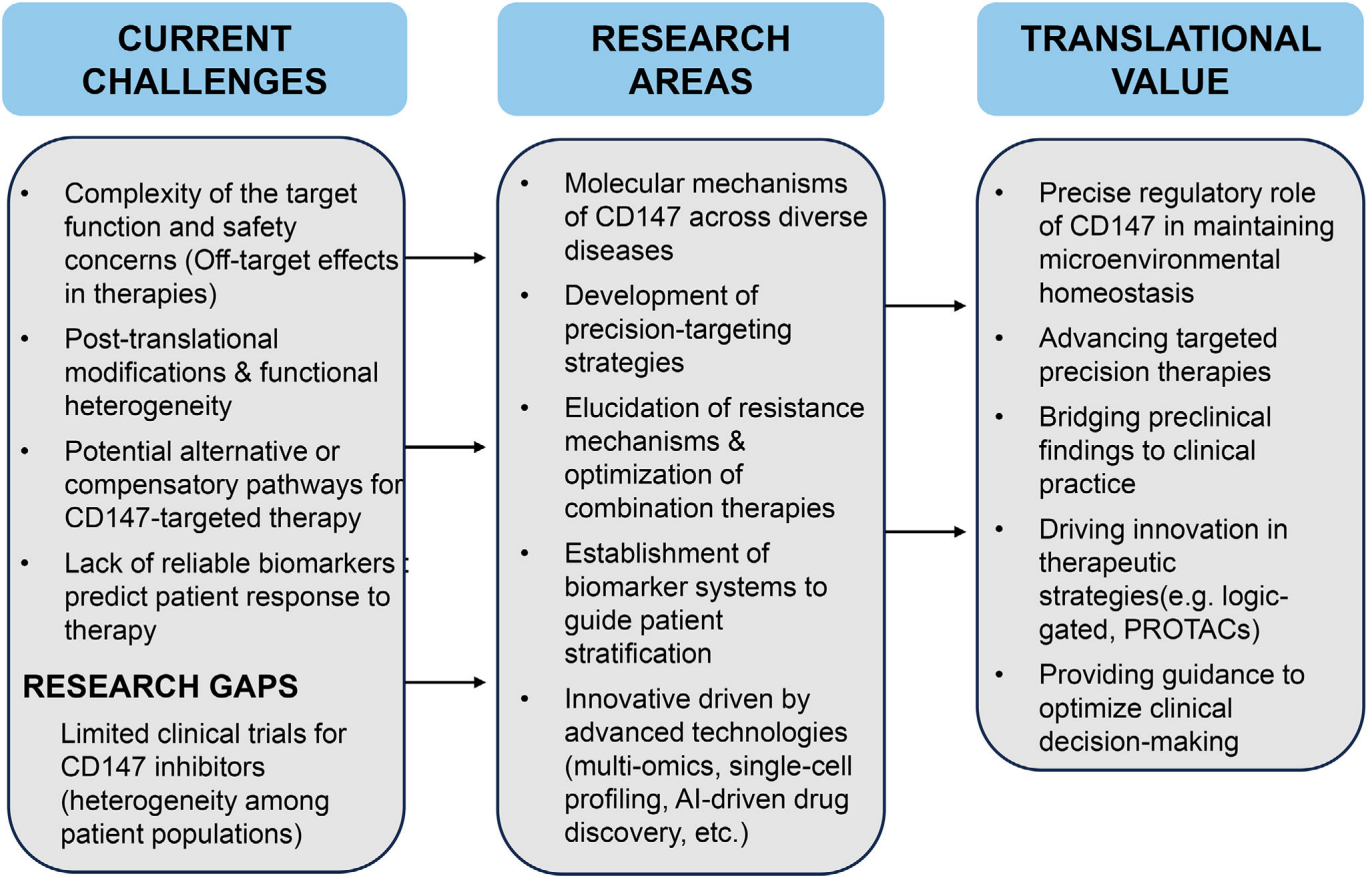
Research roadmap and translational potential of CD147. This infographic summarizes current landscape and future trajectory of CD147 translation. It contrasts current challenges (such as the “specificity paradox,” off‐target effects) with Research Priorities (molecular mechanism elucidation, biomarker discovery). Finally, it outlines the Translational Value, emphasizing the shift toward logic‐gated delivery systems, precision patient stratification, and next‐generation combination therapies to fully realize CD147's potential in clinical practice.

Ultimately, as conceptually illustrated in Figure 8, the research horizon must extend beyond the plasma membrane to subcellular and cross‐disease contexts. Emerging evidence implicating CD147 in mitochondrial bioenergetics and exosomal trafficking [13, 258] positions it as a relevant target for neurodegenerative and metabolic disorders, potentially offering novel therapeutic avenues for Alzheimer's disease. By systematically addressing these domains—structural resolution, logic‐gated delivery, resistance mapping, and subcellular exploration—this roadmap establishes a comprehensive framework to transform CD147 from a challenging target into a cornerstone of precision medicine.

## 9 Conclusion and Outlook

The scientific trajectory of CD147—from its initial identification as a “Tumor Cell‐Derived Collagenase Stimulatory Factor” to its current status as an integrative molecular hub—reflects a fundamental evolution in our understanding of cellular regulation. Functioning as a fundamental “Energy‐Structure Coupler,” CD147 orchestrates metabolic flux with morphogenetic plasticity to maintain physiological homeostasis. Over the past four decades, research has evolved from molecular identification to dissecting its multifunctional pathological roles, revealing how this machinery is hijacked to drive malignant invasion, pathogen entry, and chronic inflammation.

Despite clinical milestones such as the approval of Licartin and Meplazumab, which have validated its therapeutic potential, widespread clinical translation remains obstructed by the “Specificity Paradox.” The central challenge lies in targeting the pathological overactivity of CD147 without disrupting its broad physiological functions, a difficulty compounded by biological barriers including functional redundancy and undefined resistance mechanisms. Critical knowledge gaps persist, particularly regarding the dynamic conformational switching between its metabolic and adhesive states, as well as the functional impact of site‐specific glycosylation and genetic heterogeneity (e.g., SNPs such as rs8259) on drug response. Furthermore, emerging roles in neurobiology, mitochondrial bioenergetics, and exosomal trafficking represent critical but under‐explored frontiers.

To overcome these hurdles and fully harness the therapeutic promise of CD147, the next decade of research must pivot from broad blockade to precision modulation. This strategic shift requires resolving the structural basis of CD147's functional duality through advanced techniques like cryo‐electron microscopy and spatial omics. Concurrently, the development of microenvironment‐responsive, “logic‐gated” delivery systems will be essential to bypass the erythrocyte antigen sink and minimize systemic toxicity.

In conclusion, the translation of CD147 depends on reconciling its structural versatility with its functional specificity. By integrating deep mechanistic insights with technological innovation, the field can accelerate the maturation of CD147 from a complex biological target into a cornerstone of clinical practice. Achieving this will not only refine its application in oncology but also expand its therapeutic reach into infectious diseases and autoimmune disorders, positioning CD147 at the forefront of translational research for decades to come.

## Conflicts of Interest

The authors declare no conflicts of interest.

## Data Availability

The authors have nothing to report.
